# Thinking Twice about the Cervical Mass: A Case Report of Primary Vaginal Leiomyosarcoma and Review of the Literature

**DOI:** 10.1155/2024/1829000

**Published:** 2024-04-01

**Authors:** Caroline C. Davitt, Yingao Zhang, Anthony B. Costales

**Affiliations:** Baylor College of Medicine, Department of Obstetrics & Gynecology, Division of Gynecologic Oncology, Dan L Duncan Comprehensive Cancer Center, 7200 Cambridge St., Houston, TX 77030, USA

## Abstract

Primary vaginal leiomyosarcoma (LMS) is an unusual cause of aggressive gynecologic cancer which requires prompt surgical treatment for favorable outcomes. Definitive diagnosis and treatment render unique challenges to clinicians based on vague presentation and limited evidence for management. Here, we describe a case of vaginal LMS in a middle-aged woman with a history of cervical dysplasia found to have a proximal vaginal mass after presenting with vaginal discharge and cramping pain. The patient was diagnosed on pathologic surgical specimen and subsequently underwent definitive surgical treatment. She remains with no evidence of disease 20 months later. In our report, we emphasize the nuances of surgical management including localized source control in those desiring future fertility. Ultimately, we make recommendations for surgical treatment and surveillance based on the available published literature.

## 1. Introduction

Vaginal cancer is the second least common of gynecologic malignancies representing only 1-2% of all female pelvic cancers. Most vaginal cancers are squamous carcinomas, whereas primary vaginal sarcomas account for only 3% of vaginal cancers [1]. Presentation of these malignancies is often vague, including asymptomatic vaginal masses, dyspareunia, watery vaginal discharge, and bladder, rectal, or vaginal pain [2]. There is no consensus on management of vaginal sarcomas, with current evidence limited to case reports for vaginal leiomyosarcomas (LMS) specifically. This is in contrast to uterine sarcomas, whose prevalence among uterine malignancies is rare but whose overall incidence is significantly higher than vaginal sarcomas [3]. Uterine sarcomas thus have standardized evidenced-based management guidelines which prioritize expeditious diagnosis and effective treatment of these notoriously aggressive malignancies. Extrapolating from the higher level of evidence in this literature, surgical resection has been the mainstay of treatment for vaginal LMS. In this report, we present a relevant case that calls into question the degree of necessary surgical intervention and review published literature for treatment of vaginal sarcomas to date.

## 2. Case Presentation

**Figure 1:**
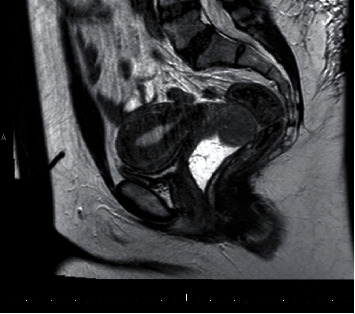
Sagittal T2 MRI of the uterus and cervix with apical vaginal mass in gel-distended vagina.

**Figure 2:**
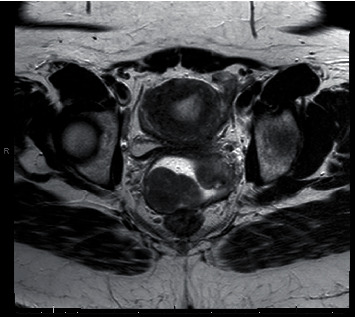
Axial T2 MRI with separate cervix (patient left) and vaginal mass (patient right).

A 40-year-old female presented to her primary gynecologist with a 3-month history of watery vaginal discharge and intermittent cramping pain. She had one vaginal delivery followed by 4 miscarriages with one dilation and curettage and no other significant medical or surgical history. Her gynecologic history includes abnormal Pap smears with a colposcopy ten years prior and normal exams since that time. On initial presentation, her primary gynecologist noted a fungating mass at her cervix. The Pap smear was negative for cytologic abnormalities, and she was referred to a gynecologic oncologist. Transvaginal ultrasound and MRI confirmed a mass in the superior vaginal fornix measuring 5.9 cm in greatest dimension without pelvic lymph node enlargement (Figure 1, sagittal; Figure 2, axial). The uterus was notable for features of adenomyosis but otherwise unremarkable. Biopsy of the vaginal mass demonstrated a cellular smooth muscle tumor with mitotically active cells and necrotic foci but without prominent atypia, leading to uncertainty surrounding its malignant potential.

Recommendation was made for diagnostic and therapeutic surgical management including hysterectomy to localize the origin of the mass. However, the patient strongly desired future fertility. Both clinical observation and scientific data suggest a correlation between feelings of depression, grief, stress, and sexual dysfunction in women who have lost their fertility due to cancer treatment [4]. Due to the uncertainty surrounding the diagnosis and the known impact of fertility loss, conservative fertility-sparing surgical management was offered given the absence of malignant confirmation. The patient underwent a cervical conization, upper vaginectomy, and vaginal mass resection. Pathology confirmed LMS with negative vaginal margins. Histologic sections demonstrated a highly mitotic spindle cell neoplasm with positive staining for desmin, smooth muscle actin, estrogen, and progesterone receptors (ER/PR). Postoperatively, a computed tomography of the chest, abdomen, and pelvis confirmed no abdominal or pelvic lymphadenopathy and was negative for any extrapelvic disease. The patient was agreeable to completion hysterectomy after additional counseling due to the aggressive nature of LMS and need to rule out a metastatic origin of LMS, most commonly from the uterus.

She then underwent an uncomplicated total laparoscopic hysterectomy with bilateral salpingectomy. Pathology from this surgery revealed proliferative endometrium with confirmed adenomyosis and with presence of benign leiomyomata. There was no evidence of malignancy in all surgical samples. She recovered well and has been without evidence of disease for 20 months as of last clinic follow-up. Informed consent was obtained from the patient to publish this case report, which was exempt from institutional review board assessment.

## 3. Discussion

Initial management of a vaginal tumor should be biopsy to establish a tissue diagnosis, as 80% of all vaginal tumors are metastatic or secondary tumors [5]. As in this case, biopsy was not sufficient nor conclusive to diagnose malignancy nor exclude uterine involvement. Primary vaginal LMS is among the rarest of primary vaginal malignancies, with less than 100 cases published in English case reports and literature reviews as of the last decade [2, 6]. When compared to squamous cell carcinomas, cohort studies have shown that vaginal sarcomas disproportionally affect younger and Black women, have larger primary tumors, and are associated with greater risk of cancer-specific and overall mortality [7].

Among vaginal sarcomas, LMS has a better overall 5-year survival rate (74.1% vs. 66.7%, *P* = 0.307) compared to other vaginal sarcomas [6]. In a United States SEER9 database study, local uterine sarcomas showed a similar overall survival (OS) rate of 74.1% at 3 years which decreased by the 5-year mark to 67.0% [8]. In early stage uterine LMS specifically (71% stage I/II of *n* = 1396 uLMS), 5-year survival rate was similar to that of all uterine sarcomas at 66.0% [9]. Surgery is the mainstay of treatment for both uterine and vaginal sarcomas, with 87.7% of gynecologic sarcomas receiving surgery as initial treatment and 39.9% receiving surgery as their only treatment [8]. In 66 cases of vaginal LMS, zero patients survived beyond 36 months with primary chemotherapy or radiation treatment whereas a 5-year survival rate of 57% was noted in those treated primarily with surgery [10]. With a young and otherwise healthy patient desiring future fertility, the question then becomes just how much tissue should be resected?

Given the low incidence of vaginal sarcomas, there is limited data to provide evidence-based recommendations for extent of surgical resection. For the last thirty years, surgical resection for vaginal sarcomas has ranged from wide local excision to posterior pelvic exenteration—with grade at diagnosis initially being the most important predictor of outcome and high-grade disease having higher rates of recurrence and mortality [11]. There is some evidence for radical surgery being associated with better prognosis in vaginal sarcoma as one case series (*n* = 11 surgically managed, 8 received simple resection) revealed all 8 patients undergoing simple resection despite stage I disease experienced tumor recurrence with a median time to first recurrence of 13.6 months [6]. This is corroborated by reports revealing recurrence of vLMS at site of prior wide local resection in 11 months with subsequent complete resection of recurrent disease as well as surrounding organs, albeit with adjuvant treatment, leading to at least 29 months of disease absence in initial high-grade disease [2]. In a recent review of the role of surgery in gynecologic sarcomas, fertility-sparing surgery is specifically discussed in the context of uLMS as “an extremely critical subject” that “lacks strong evidence” and should be considered an “experimental procedure” until more evidence can be provided [12]. This paper recommends the use of uLMS to guide that of cervical sarcomas due to limited evidence and does not specifically comment on vaginal sarcomas beyond rhabdomyosarcoma and endometrial stromal sarcoma (ESS) [12]. ESS, specifically early-stage disease, of the uterus has been associated with 50% successful conception rate following fertility-sparing surgery. Recurrence rate of 50% after median follow-up of 15 months with only 1 death of disease was found, underscoring the importance of patient-centered counseling and shared decision-making [13]. The extremely low incidence of vLMS has led to a lack of consensus regarding surgical management recommendations, and as such, we recommend degree of surgical management be individualized to patient priorities: weighing both the risks of a progression of an occult intrauterine primary malignancy or local recurrence of vaginal primary as well as the benefits of preserving fertility, especially in a young, nulliparous woman.

In poor surgical candidates, next-generation sequencing may be warranted given the advent of targeted chemotherapeutics and new biologic agents. Adjuvant chemotherapy has been used minimally in patients with vaginal LMS and demonstrated marginal efficacy in improving local and distant recurrence [6]. However, hormone-modulating agents can be considered in ER/PR-positive vaginal LMS, as extrapolation from phase 2 clinical trials in uterine sarcomas demonstrates an improvement in progression-free survival [14]. Similarly for radiotherapy, data specific to vaginal LMS is sparse and warrants extrapolation from studies on uterine sarcomas and other vaginal malignancies [5]. Unfortunately, radiotherapy in uterine LMS has not been shown to improve local control nor survival compared to observation alone [7, 15]. Thus, radiotherapy likely plays a minimal role in the primary treatment of vaginal LMS.

Overall, postsurgical surveillance is an essential aspect of vaginal LMS. Risk for recurrence in vaginal sarcoma is high, most commonly with local recurrence vaginally, found in 69.2% of recurrences in a series of 15 patients [6]. Surveillance should involve regular, short-interval clinic visits with comprehensive history and physical exam as well as imaging studies. We recommend history and physical exam every 3-4 months and CT or MRI of the abdomen and pelvis every 3-6 months for the first 2-3 years following diagnosis, which can then be spaced to every 6-12 months for the following 2 years based on surveillance recommendations for uterine LMS [3]. Depending on histologic grade and initial stage, annual to biannual imaging can be used for surveillance for recurrence for an additional 5 years [3]. If widespread metastasis is suspected, FDG-PET scans can be considered to better characterize FDG-avid lesions. Given the propensity for hematogenous metastatic spread of LMS, hepatic and pulmonary metastases are common secondary sites for recurrence. One report from our institution showed greater than 2 years of disease-free survival after thoracotomy for isolated recurrent vaginal LMS which can be considered in extremely select cases [16].

The limitations of this study include the nature of a case report which cannot infer causality of favorable disease-free interval outcomes from uncontrolled observational intervention. Treatment was retrospectively reviewed and nonrandomized. Results may not be generalizable to all populations with vaginal sarcomas, rather focused on young, fertility-desiring individuals. Additionally, it must be emphasized that fertility preservation in a disease with such a poor prognosis is not without substantial risk. Due to its rarity, it remains unknown what impact fertility preservation has on vLMS recurrence risk. Effective patient-physician communication is both required and essential for the patient to make an informed decision regarding which risks they are willing to accept.

In conclusion, this case describes a case of early-stage isolated vLMS managed with definitive surgery in a young premenopausal patient. Even though a hysterectomy was ultimately performed, her original request for fertility-sparing treatment is thought-provoking for management strategies of this uncommon malignancy. The rare incidence of this disease with the scarcity of reported cases in the current literature limits standardization of care outside of extrapolation from related malignancy such as uLMS. Each case necessitates an individualized approach to management and is reasonable to include discussion of a fertility-sparing option in carefully selected patients. We continue to encourage the publication of similar cases to contribute to the breadth and depth of knowledge on this rare cancer to develop cohesive treatment recommendations for patients.

## Data Availability

The clinical case data used to support the findings of this study are included within the article.
